# SVIP reduces IGFBP-2 expression and inhibits glioblastoma progression via stabilizing PTEN

**DOI:** 10.1038/s41420-024-02130-z

**Published:** 2024-08-13

**Authors:** Zixuan Wang, Xiaolong Qiao, Yinan Chen, Nan Peng, Chaoshi Niu, Yang Wang, Cong Li, Zengchun Hu, Caihua Zhang, Chuandong Cheng

**Affiliations:** 1 Department of Neurosurgery, Centre for Leading Medicine and Advanced Technologies of IHM, The First Affiliated Hospital of USTC, Division of Life Sciences and Medicine, University of Science and Technology of China, Hefei, Anhui 230001 China; 2 Dalian Medical University, Dalian, Liaoning 116000 China; 3 Anhui University of Science and Technology, Huainan, Anhui 232001 China; 4 Department of Neurosurgery, 2nd Affiliated Hospital of Dalian Medical University, Dalian, Liaoning 116023 China

**Keywords:** Ubiquitylation, CNS cancer, Ubiquitylated proteins

## Abstract

Glioblastoma (GBM) presents significant challenges due to its invasive nature and genetic heterogeneity. In this study, we investigated the impact of Small VCP/P97-Interacting Protein (SVIP) on GBM progression. Our results revealed elevated expression of Insulin-like Growth Factor Binding Protein 2 (IGFBP-2) and STIP1 homology and U-box containing protein 1 (STUB1), coupled with reduced SVIP levels in GBM samples. Notably, high IGFBP-2 expression correlated with poor prognosis. Mechanistically, SVIP competitively inhibited STUB1, selectively binding to VCP/p97, thereby reducing PTEN degradation. This SVIP-mediated regulation exerted influence on the PTEN/PI3K/AKT/mTOR pathway, leading to the suppression of GBM progression. Co-localization experiments demonstrated that SVIP hindered PTEN ubiquitination and degradation by outcompeting STUB1 for VCP/p97 binding. Moreover, SVIP overexpression resulted in reduced activation of AKT/mTOR signaling and facilitated autophagy. In vivo experiments using a GBM xenograft model substantiated the tumor-suppressive effects of SVIP, evident by suppressed tumor growth, decreased IGFBP-2 expression, and improved survival rates. Collectively, our findings underscore the functional significance of SVIP in GBM progression. By inhibiting STUB1 and stabilizing PTEN, SVIP modulates the expression of IGFBP-2 and attenuates the activation of the PI3K/AKT/mTOR pathway, thereby emerging as a promising therapeutic target for GBM treatment.

## Introduction

The high invasiveness, genetic heterogeneity and the physical isolation of blood brain barrier (BBB) are challenges to treat glioblastoma multiform (GBM) [1, 2]. Therefore, it is important to understand the mechanisms and find new targets related to the progress of GBM.

PTEN (phosphatase and tensin homolog on chromosome 10) is widely recognized as a prominent tumor suppressor. The presence and activation of PTEN in the cytoplasmic membrane is essential to ensure the control of PI3K signal transduction. As a doubly specific lipid and protein phosphatase, PTEN dephosphorylates the 3 ‘group of PIP3 effectively, thereby terminating signal transduction to AKT and other PIP3 effect targets. Therefore, PTEN/PI3K is a key functional axis that regulates the activation state of multiple proto-cancer signals in a coordinated manner, which can be cleared during tumorigenesis or used by cancer cells to overgrow [3, 4]. Interestingly it is mutated in almost all major human cancer types, and the mutation frequency is second only to that of p53 [5]. Unlike classical tumor suppressor genes, which require complete inactivation to induce cancer, partial loss of PTEN function can have a dramatic effect on tumor occurrence and progression [6].

STUB1 (STIP1 homology and U-box containing protein 1) or CHIP (Carboxy terminus of Hsp70-interacting protein) has U-box-dependent E3 ubiquitin ligase activity, which can act as a molecular chaperone to degrade misfolded proteins [7, 8]. It also contains three tandem tetrapeptides (TPR), which interact with molecular chaperones Hsp70 and Hsp90; thus, ubiquitinate the chaperone binding substrate to maintain intracellular protein homeostasis [9]. Previous studies have demonstrated that STUB1/CHIP is one of the E3 ubiquitin ligases of PTEN [10, 11].

Small VCP/ p97-Interacting proteins (SVIP) were found as the ligand of VCP/p97 via a VCP/p97 interacting motif (VIM) [12, 13]. Although VCP/p97 does not contain the binding site to ubiquitinated proteins, it helps degrading protein substrates by binding to chaperone proteins containing ubiquitination binding regions. It has been reported that SVIP competitively bind VCP/p97 to some E3 ubiquitin ligases via VIM, for example, gp78 and Hrd1, and thereby inhibits ERAD. Relevant studies have shown that not only SVIP containing VIM, but also STUB1/CHIP containing VCP/p97 binding motif (VBM) can interact with VCP/p97 and play different biological functions [14, 15]. Moreover, the α-helical surface of VIM structure forms more positive charge than that of VBM structure, which is more favorable for binding to VCP/p97 protein [16–18]. Therefore, SVIP, theoretically, is more easily combined with VCP/p97, and can play a competitive inhibitory effect on STUB1/CHIP.

Insulin-like growth factor binding protein 2 (IGFBP-2) is a key member of the IGFBP family [19, 20]. It has been previously reported that IGFBP-2 is highly expressed in many tumors, including GBM, and is closely associated with cell proliferation, migration, invasion, angiogenesis, epithelial-mesenchymal transformation (EMT) and so on [21–24]. In glioma, surprisingly, IGFBP-2 overexpression is associated with PTEN deficiency. IGFBP-2 is negatively regulated by PTEN and its expression can indicate the activation of PI3K/AKT pathway and the status of PTEN [25, 26]. Meanwhile, IGFBP-2 is an exocrine protein that is detectable in tissue and blood. The level of IGFBP-2 in tumor tissue and plasma of patients with early glioma has been significantly increased. Also, the recurrence period and overall survival of patients with high expression of IGFBP-2 in the tumor have been significantly shortened [27, 28].

In our study, SVIP expression levels were decreased and STUB1/CHIP expression levels were increased in glioblastoma, so that the two were out of balance compared with non-tumor tissues. However, overexpression of SVIP can exert its competitive advantage. By competitively inhibiting STUB1, SVIP preferentially binds to VCP/p97 and decrease the degradation of PTEN. In this way, SVIP exerts its regulatory effect on PI3K/AKT/mTOR pathway, ultimately alters the expression of IGFBP-2 and arrests the progression of GBM.

## Results

### IGFBP-2 exhibits high expression levels in both glioblastoma cells and glioblastoma samples

**Fig. 1:**
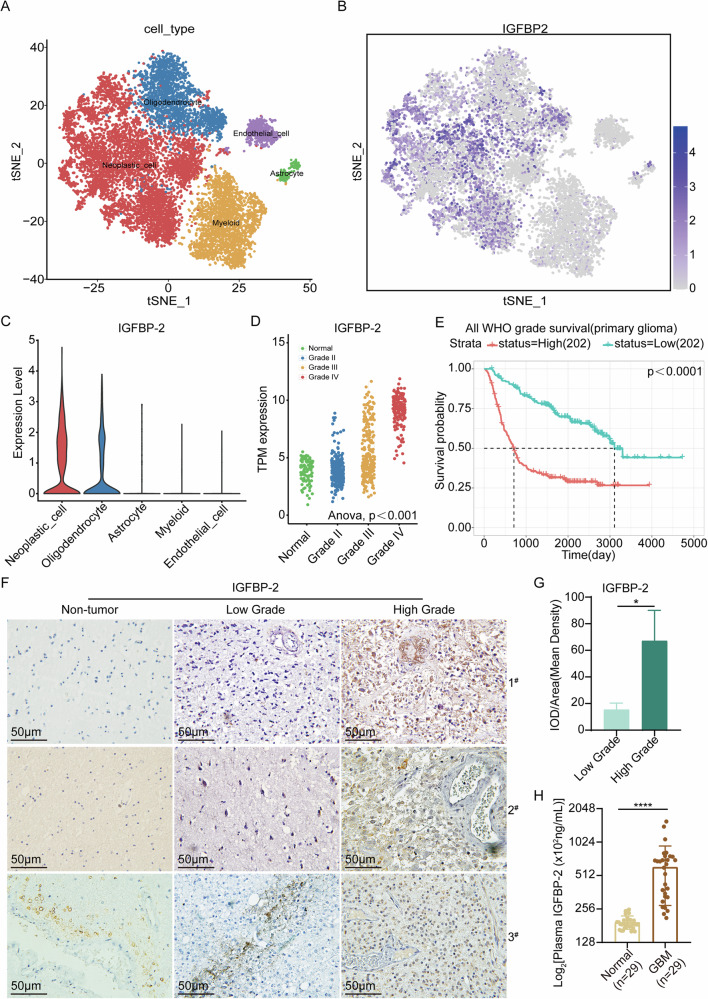
IGFBP-2 Expression Levels in Glioblastoma Cells and Tissue Samples. **A**–**C** Analyzing the expression of IGFBP-2 using single-cell sequencing data from the GSE138794 database. **D** CGGA database was used to analyse the expression levels of IGFBP-2 in different grades of gliomas. **E** CGGA database analysis of overall survival in patients with different expression levels of IGFBP-2. High (*n* = 202) and low (n = 202) levels of IGFBP-2, *P* < 0.05. **F** IHC was conducted to assess the expression levels of IGFBP-2 in human non-tumor brain tissue, low-grade (WHO I and II) glioma tissue, and high-grade (WHO III and IV) glioma tissue samples. All images were captured at 40× magnification. **G** Quantitative analysis was conducted on the immunohistochemical staining of low-grade and high-grade gliomas. **H** Plasma IGFBP-2 expression levels were measured by ELISA in a cohort of 58 subjects, including 29 healthy controls and 29 GBM patients. Statistical analysis: data were quantified as mean ± SD, *n* ≥ 3, two tailed student’s *t* test, *P* < 0.05, *; *P* < 0.0001, ****.

The high expression of IGFBP-2 is an indicator of poor prognosis in GBM patients. In accordance with the methodology and parameter settings of a previous study, we conducted single-cell analysis to examine IGFBP-2 expression in tumor cells, utilizing scRNA-Seq data from 9 patients in the GSE138794 dataset [29]. Our analysis unveiled a notable overexpression of IGFBP-2 in tumor cells (Fig. 1A–C). In the CGGA (Chinese Glioma Genome Atlas) database, we observed a significant upregulation of IGFBP-2 expression levels with the increasing WHO grading of gliomas (Fig. 1D). We also found that high expression of IGFBP-2 was associated with poor prognosis according to CGGA database (Fig. 1E). We performed IGFBP-2 expression level detection in clinical tissue samples. Our findings, consistent with bioinformatics analysis, demonstrate significant upregulation of IGFBP-2 in high-grade glioma tissue samples (Fig. 1F, G). To confirm the consistency of IGFBP-2 levels in plasma and tumors, we performed ELISA assays to measure plasma IGFBP-2 levels in both non-tumor and GBM patient groups. The results showed a significant elevation in plasma IGFBP-2 levels among GBM patients compared to non-tumor controls, consistent with the results obtained from IHC (Fig. 1H).

### IGFBP-2 influences the proliferation and migration of GBM cells

**Fig. 2:**
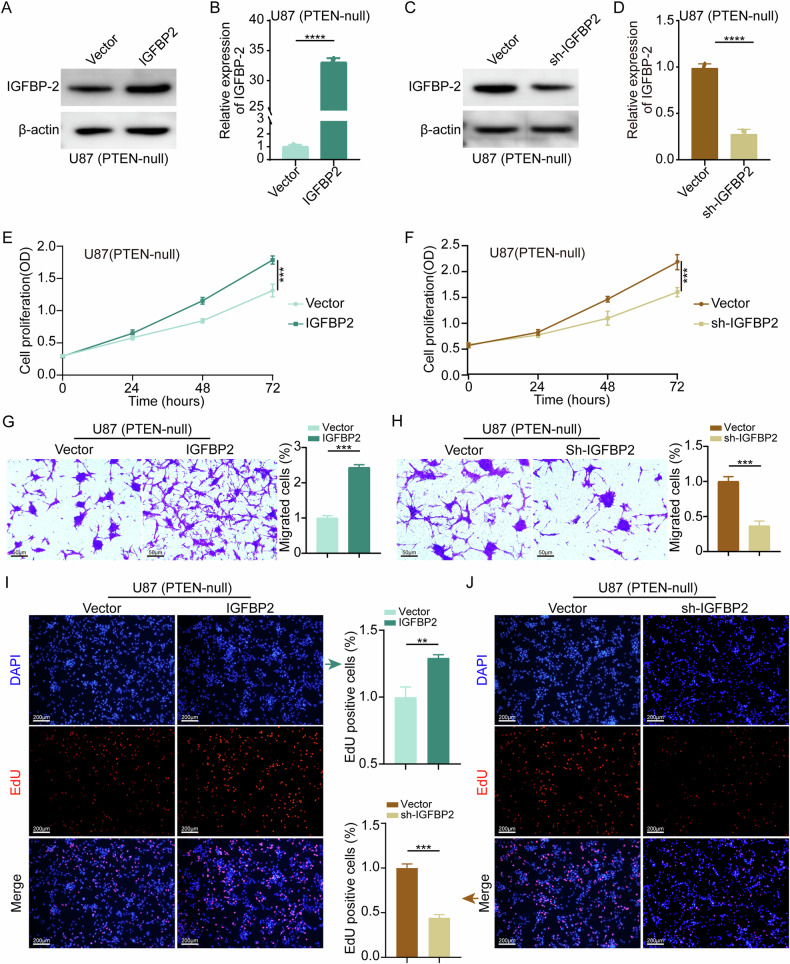
Impact of IGFBP-2 on Proliferation and Migration of GBM Cells. **A**–**D** The efficiency of IGFBP-2 overexpression and knockdown was evaluated using Western blot and qRT-PCR techniques. **E**, **F** In U87-MG cells, IGFBP-2 was respectively overexpressed or knocked down, followed by cell viability assessment using the CCK-8 assay. **G**, **H** In U87-MG cells, IGFBP-2 was respectively overexpressed and knocked down, followed by the assessment of cell migration ability using the Transwell assay. **I**, **J** In U87-MG cells, IGFBP-2 was respectively overexpressed and knocked down, followed by the assessment of cell proliferation using EdU labeling. Statistical analysis: data were quantified as mean ± SD, *n* ≥ 3, two tailed student’s *t* test, *P* < 0.01, **; *P* < 0.001, ***; *P* < 0.0001, ****.

We verified whether alterations in IGFBP-2 expression levels would affect the biological functions of GBM cells. We respectively overexpressed and knocked down IGFBP-2 in U87-MG (PTEN-null) and validated their efficiency using Western blot and qRT-PCR (Fig. 2A–D). Through CCK-8 cell viability assays, we found that the viability of U87-MG cells significantly increased with higher levels of IGFBP-2 expression, and conversely decreased with lower levels of IGFBP-2 expression (Fig. 2E, F). Through Transwell cell migration assay, we observed a significant enhancement in the migratory capacity of U87-MG cells with increased levels of IGFBP-2 expression. Conversely, decreased expression levels of IGFBP-2 resulted in weakened migratory capacity (Fig. 2G, H). In the EdU cell proliferation assays, it was evident that higher expression levels of IGFBP-2 led to a significant increase in the proliferation rate of U87-MG cells. Conversely, decreased expression levels of IGFBP-2 resulted in reduced proliferation of U87-MG cells (Fig. 2I, J).

### STUB1 and SVIP regulate the expression of PTEN and IGFBP-2, as well as the proliferation and migration of GBM cells

**Fig. 3:**
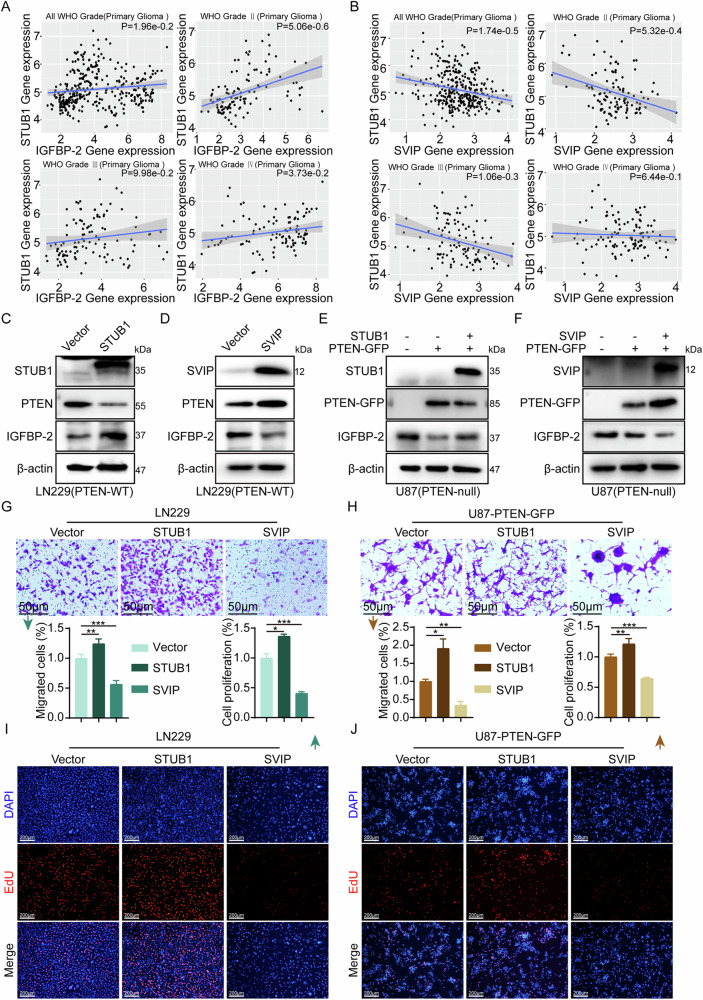
STUB1 and SVIP Regulate the Expression of PTEN and IGFBP-2 and the Biological Functions of GBM Cells. **A** The CGGA database was utilized to analyze the correlation between the expression levels of *STUB1* and *IGFBP-2* across various grades of gliomas. **B** The CGGA database was utilized to analyze the correlation between the expression levels of *STUB1* and *SVIP* across various grades of gliomas. **C**, **D** In LN229 cells, transfection was performed with STUB1 or SVIP overexpression plasmids separately. Subsequently, Western blot analysis was conducted to assess the expression levels of STUB1, SVIP, PTEN, and IGFBP-2. **E** U87-MG cells were co-transfected with PTEN-GFP overexpression plasmid and empty vector, PTEN-GFP and STUB1 overexpression plasmids, respectively. Western blot analysis was conducted to evaluate the protein expression levels of STUB1, PTEN, and IGFBP-2 in the U87-MG cells. **F** U87-MG cells were co-transfected with PTEN-GFP overexpression plasmid and empty vector, PTEN-GFP and SVIP overexpression plasmids, respectively. Western blot analysis was conducted to evaluate the protein expression levels of SVIP, PTEN, and IGFBP-2 in the U87-MG cells. **G**, **I** In LN229 cells, plasmid transfection was conducted as described above. The migration ability of the cells was evaluated using the Transwell assay, while the proliferation capacity was assessed through EdU staining. **H**, **J** In U87-MG cells, plasmid transfection was conducted as described above. The migration ability of the cells was evaluated using the Transwell assay, while the proliferation capacity was assessed through EdU staining. Statistical analysis: data were quantified as mean ± SD, n ≥ 3, two tailed student’s t test, *P*＜0.05, *; *P*＜0.01,**; *P*＜0.001,***.

In previous studies, IGFBP-2 has been utilized as an indicator of PTEN status and the activation of the PI3K/AKT pathway, while STUB1 is known as the E3 ubiquitin ligase of PTEN. Therefore, we analyzed the correlation between STUB1 and IGFBP-2 in the CGGA database, and observed a positive correlation between them in gliomas (Fig. 3A). As a potential competitor of STUB1, the expression of SVIP is negatively correlated with that of STUB1 in all WHO grades (Fig. 3B). To investigate the influence of SVIP or STUB1 on PTEN and IGFBP-2, we transfected STUB1 or SVIP expression plasmids into LN229 (PTEN-WT) cells. In U87-MG (PTEN-null) cells, we transfected wild-type PTEN plasmids fused with GFP tags, while concurrently overexpressing STUB1 or SVIP individually. We observed that overexpression of STUB1 in GBM cells resulted in decreased protein levels of PTEN and increased expression of IGFBP-2 (Fig. 3C, E). The trend was opposite when SVIP was overexpressed (Fig. 3D, F). Subsequently, we investigated the impact of STUB1 or SVIP expression on GBM proliferation and migration. We observed that overexpression of STUB1 promoted the proliferation and migration of LN229 and U87-MG cells, whereas overexpression of SVIP markedly reduced the proliferation and migration ability of the cells (Fig. 3G–J, Supplementary Fig. 1A–D). These findings suggest that both SVIP and STUB1 are capable of regulating the expression levels of PTEN and IGFBP-2, thereby influencing the proliferation and migration abilities of GBM cells.

### SVIP prevents PTEN degradation by competing with STUB1 for VCP/p97 binding

**Fig. 4:**
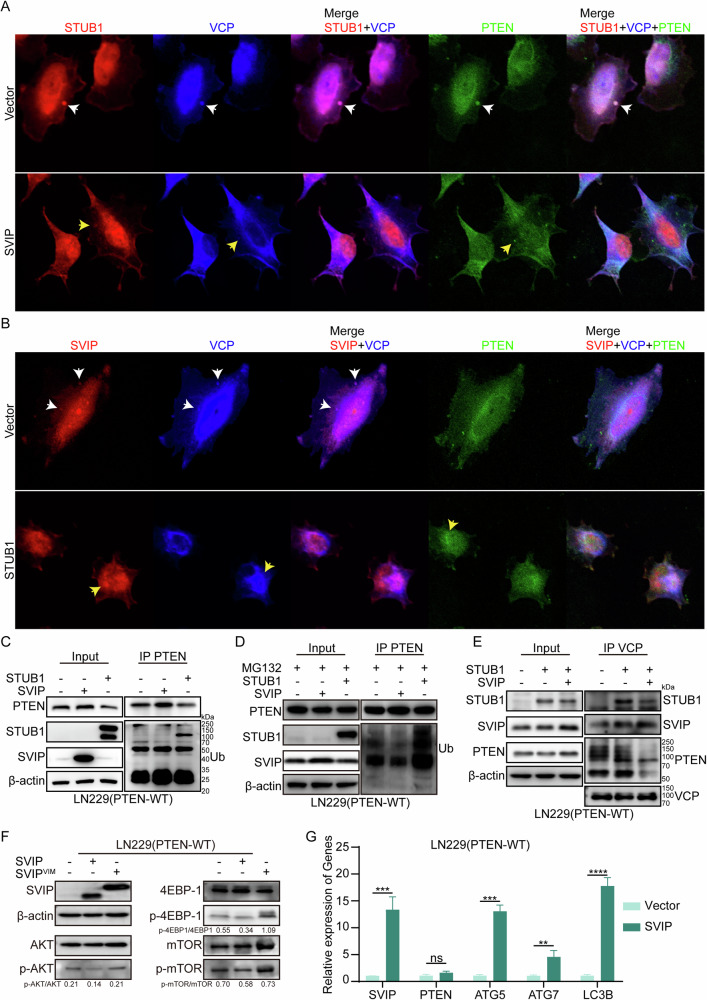
SVIP Inhibits PTEN Ubiquitination by Competing with STUB1. **A** In LN229 cells, transfection was performed with plasmids overexpressing SVIP and empty vector separately. Immunofluorescence staining was used to label STUB1, VCP, and PTEN. The localization of STUB1, VCP, and PTEN was examined using fluorescence microscopy. **B** In LN229 cells, transfection was performed with plasmids overexpressing STUB1 and empty vector separately. Immunofluorescence staining was used to label SVIP, VCP, and PTEN. The localization of SVIP, VCP, and PTEN was examined using fluorescence microscopy. **C**, **D** In LN229 cells, plasmids overexpressing STUB1 and SVIP were separately transfected. After 48 h of transfection, cells were treated with or without MG132 (50 µM, TOPSCIENCE, China) for 2 h. Immunoprecipitation (IP) was performed using an anti-PTEN antibody, followed by immunoblotting (IB) with an anti-Ub antibody to assess the binding between PTEN and Ub. **E** In LN229 cells, co-transfection was conducted using either the STUB1 overexpression plasmid and empty vector, or the STUB1 and SVIP overexpression plasmid. Immunoprecipitation (IP) was carried out using an anti-VCP antibody, followed by immunoblotting (IB) with anti-STUB1, anti-SVIP, and anti-PTEN antibodies, respectively, to detect the interaction between VCP and PTEN. **F** Transfect LN229 cells with SVIP and SVIP^VIM^ overexpression plasmids, then assess Akt, mTOR, and 4EBP-1 expression and phosphorylation levels via Western blot. **G** qRT-PCR was used to detect the mRNA levels of *SVIP*, *PTEN*, *ATG5*, *ATG7*, and *LC3B* after SVIP overexpression plasmids were transfected in LN229 cells. Statistical analysis: data were quantified as mean ± SD, *n* ≥ 3, two tailed student’s *t* test, *P* < 0.01, **; *P* < 0.001, ***; *P* < 0.0001, ****; ns, no significance.

To assess the co-localization of STUB1, SVIP, and PTEN, we initially transfected LN229 cells with SVIP overexpression plasmids. Following this, immunofluorescence labeling was performed to separately label STUB1, VCP/p97, and PTEN. We observed that under normal conditions, STUB1 co-localized with both VCP/p97 and PTEN (indicated by white arrows). However, upon SVIP overexpression, the co-localization of STUB1 with PTEN notably decreased, and in some instances, no co-localization was detected (indicated by yellow arrows) (Fig. 4A). We further overexpressed STUB1 in LN229 cells and conducted immunofluorescence labeling of SVIP, VCP/p97, and PTEN. In the absence of STUB1 overexpression, we observed co-localization of SVIP with VCP/p97 but did not detect co-localization with PTEN (indicated by white arrows). However, upon STUB1 overexpression, the co-localization of SVIP with VCP/p97 decreased, and co-localization with PTEN remained absent (indicated by yellow arrows) (Fig. 4B). To validate the impact of STUB1 and SVIP on PTEN ubiquitination, we transfected vector control, STUB1, and SVIP overexpression plasmids into LN229 (PTEN-WT) cells, respectively. The results demonstrated that the PTEN expression level (Fig. 4C) remained consistent with that shown in Fig. 3. Upon PTEN precipitation using anti-PTEN antibody, the ubiquitinated PTEN level was notably higher in cells overexpressing STUB1 compared to those overexpressing SVIP (Fig. 4C). After inhibiting the proteasome with MG132, the protein levels of PTEN were sustained. The ubiquitination status remained consistent with previous observations (Fig. 4D). In order to illustrate the competition between SVIP and STUB1 for forming a VCP-PTEN complex, we transfected STUB1-expressing plasmid in combination with control vector and SVIP-expressing plasmid in LN229 cells, respectively. The immunoprecipitation results showed that when STUB1 was overexpressed, VCP/p97 complex contained more STUB1 and PTEN (and ubiquitinated PTEN) than control cells. Whereas, SVIP overexpression significantly reduced the quantity of STUB1 and PTEN in the VCP/p97 complex in comparison with that of STUB1 overexpressed cells (Fig. 4E). Next, we verified SVIP-VCP interaction could affect AKT/mTOR signaling via relief of PETN. We overexpressed SVIP, SVIP^VIM^ (a mutant that loss of VCP/p97 binding) and vector control in LN229 cells. Western-blot results showed that overexpression of wildtype SVIP reduced obviously the phosphorylation of AKT, mTOR and downstream protein 4EBP-1. However, destructed SVIP-VCP interaction by overexpressing of the VIM mutant of SVIP (SVIP^VIM^), the phosphorylation of AKT and mTOR were not affected (Fig. 4F). In addition, to exclude the possibility that PTEN expression was regulated on transcriptional level, mRNA expression of related genes was analyzed by qRT-PCR. Previous studies have shown that endogenous SVIP regulates autophagy by promoting LC3 lipidation, enhancing p62 expression, and sequestering polyubiquitinated proteins within autophagosomes. Knockdown of SVIP decreases LC3 lipidation and reduces p62 mRNA and protein levels [30–32]. Therefore, autophagy-related genes (including ATG5, ATG7, and LC3B) were selected as positive controls in response to SVIP overexpression. We found that overexpression of SVIP activated autophagy, resulting in increased transcription of autophagy related genes, while transcription levels of PTEN did not change significantly (Fig. 4G). These results demonstrated that SVIP competed with STUB1 for VCP/p97 binding, preventing PTEN ubiquitinated degradation at protein level, and played a regulatory role in the downstream pathways.

### SVIP regulates AKT/mTOR pathway via wild-type PTEN

**Fig. 5:**
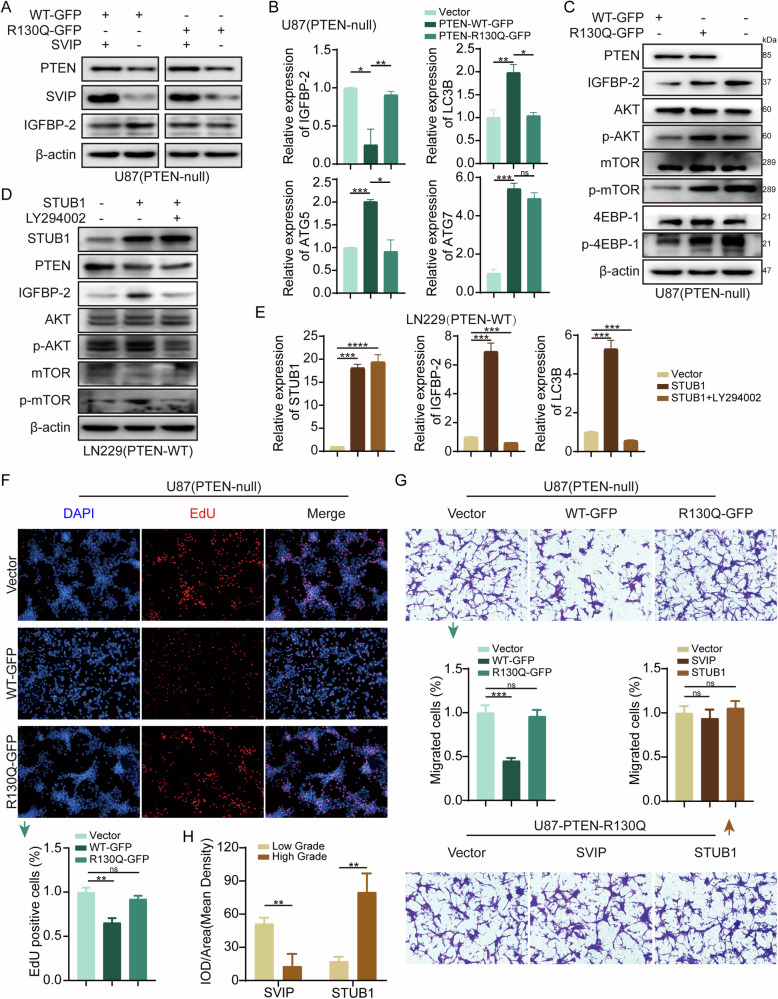
SVIP Regulates AKT/mTOR Pathway via PTEN^wt^. **A** Co-transfection experiments were conducted in U87-MG cells with PTEN-WT-GFP or PTEN-R130Q-GFP overexpression plasmids, along with empty vectors or SVIP overexpression plasmids. Subsequently, Western blot analysis was employed to assess the protein levels of SVIP, PTEN, and IGFBP-2. **B** PTEN-WT-GFP and PTEN-R130Q-GFP overexpression plasmids were transfected into U87-MG cells, and the mRNA expression levels of *IGFBP-2*, *LC3B*, *ATG7*, and *ATG5* were assessed using qRT-PCR. **C** PTEN-WT-GFP and PTEN-R130Q-GFP overexpression plasmids were transfected into U87-MG cells, and the protein expression levels and phosphorylation levels of Akt, mTOR, and 4EBP-1 were assessed by Western blot. **D** The STUB1 overexpression plasmid was transfected into LN229 cells, followed by treatment with or without the LY294002 inhibitor (20 μM, TOPSCIENCE, China). Subsequently, the expression levels of STUB1 and PTEN, as well as the phosphorylation levels of AKT and mTOR, were assessed by Western blot. **E** The STUB1 overexpression plasmid was transfected into LN229 cells, with or without the LY294002 inhibitor. Subsequently, the expression levels of *STUB1*, *IGFBP-2*, and *LC3B* were assessed by qRT-PCR. **F** EdU cell proliferation staining was performed to assess the proliferation of U87-MG cells transfected with PTEN-WT-GFP and PTEN-R130Q-GFP overexpression plasmids separately. **G** Transwell assays were performed to assess the migration of U87-MG cells transfected with PTEN-WT-GFP and PTEN-R130Q-GFP overexpression plasmids individually. Following transfection of PTEN-R130Q-GFP overexpression plasmid into U87-MG cells, transwell assays were conducted to evaluate cell migration after subsequent transfection with STUB1, SVIP, and empty vector. **H** Immunohistochemical staining was performed for quantitative analysis of SVIP and STUB1 expression in the tissues. Statistical analysis: data were quantified as mean ± SD, *n* ≥ 3, two tailed student’s *t* test, *P* < 0.05, *; *P* < 0.01, **; *P* < 0.001, ***; *P* < 0.0001, ****; ns, no significance.

Former results have shown that STUB1 and SVIP regulates the degradation of PTEN and they may play regulatory roles in AKT and mTOR signaling. We wondered the status of PTEN and its impact on AKT/mTOR pathway and GBM progression. The function of SVIP was observed in U87 cells that expressed PTEN-WT-GFP or PTEN-R130Q-GFP (PTEN-R130Q mutation loss of phosphorylase activity). WB results showed that after overexpressing SVIP, PTEN expression increased in both wild-type and mutant PTEN-expressing cells. However, only PTEN^wt^ decreased IGFBP-2 expression (Fig. 5A). Next, qRT-PCR results showed that IGFBP-2 expression on mRNA level was significantly down-regulated by only PTEN^wt^ (Fig. 5B). The mRNA expression levels of positive control genes, LC3B, ATG5 and ATG7 in the downstream of mTOR were significantly increased in the condition of PTEN overexpression compared with vector control (Fig. 5B). These results provided a clue that PTEN regulates IGFBP-2 expression via AKT/mTOR pathway in GBM cells. The following experiments showed that the phosphorylation of AKT, mTOR, downstream 4EBP-1 and the expression of IGFBP-2 decreased in PTEN^wt^ overexpressing cells compared with control cells, while PTEN-R130Q had few effects compared with control (Fig. 5C). Another experiment also proved that STUB1 induced IGFBP-2 expression on transcriptional level could be inhibited by a PI3K inhibitor, LY294002, in LN229 cells (Fig. 5D, E). We also investigated the effect of PTEN wild type or mutant protein on malignant phenotype of GBM cells. Cellular functional experiments showed that overexpression of PTEN^wt^ significantly reduced the migration and proliferation ability of GBM cells, while PTEN-R130Q had no significant difference from vector control (Fig. 5F, G, Supplementary Fig. 2A, C). We overexpressed STUB1 and SVIP in U87-PTEN-R130Q cells, respectively, and found that STUB1 or SVIP had no significant effect on cell migration and proliferation in the absence of PTEN phosphatase activity (Fig. 5G, Supplementary Fig. 2B, D). At the tissue level, we conducted an examination of SVIP and STUB1 expression. In non-tumor brain tissue, both STUB1 and SVIP were expressed in similar regions. Conversely, in high-grade glioma tissues, there was a notable upregulation in STUB1 expression, whereas SVIP expression exhibited a decline (Fig. 5H, Supplementary Fig. 3A–C). These data suggest that STUB1 or SVIP have regulatory effects on both wildtype and mutant PTEN protein expression, but SVIP showed an impact on IGFBP-2 expression and cellular function mediated by only wildtype PTEN.

### Overexpression of SVIP can delay the progression of GBM

**Fig. 6:**
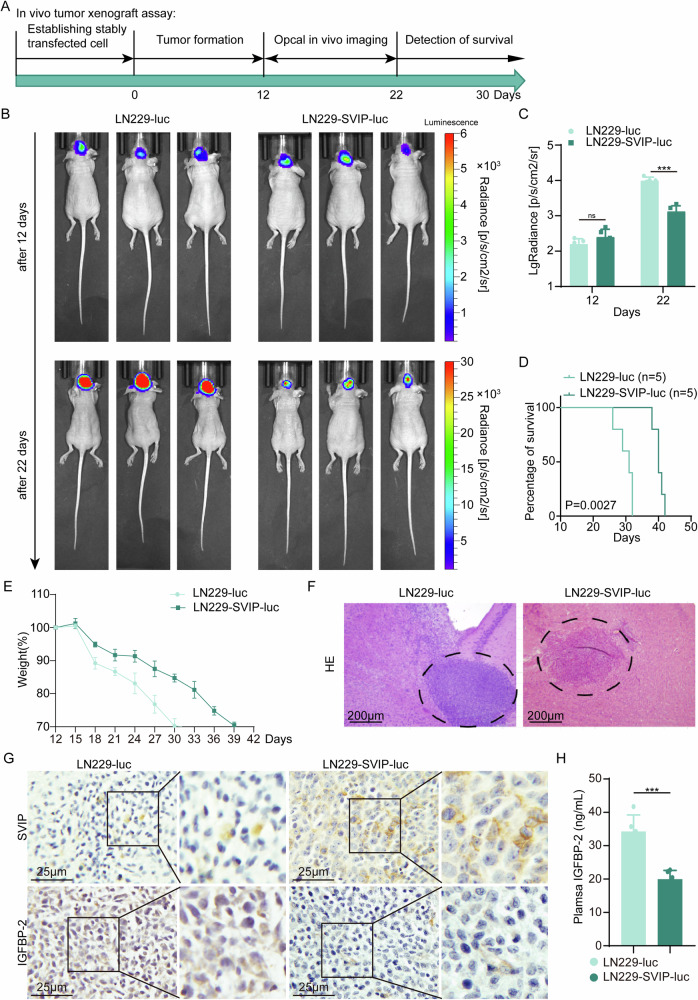
In Vivo Validation of SVIP Expression Effects on GBM. **A** The schematic diagram of in vivo tumor implantation in nude mice. **B** Bioluminescence images of nude mice. **C** Quantification of bioluminescent signal intensities in nude mice. **D** The Kaplan‒Meier survival curve of nude mice. **E** Statistics of body weight alteration curves in nude mice. **F** HE staining of murine brain paraffin sections showing tumorigenesis. **G** IHC was used to detect the expression of SVIP and IGFBP-2 in murine brain paraffin sections. **H** The plasma IGFBP-2 expression levels in experimental and control mice were detected by ELISA. Statistical analysis: data were quantified as mean ± SD, *n* ≥ 3, two tailed student’s *t* test, *P* < 0.001, ***; ns, no significance.

To investigate the impact of SVIP on GBM growth and its regulation of IGFBP-2 expression in vivo, we generated stable cell lines LN229-SVIP-luc and LN229-luc expressing SVIP-luc and luciferase, respectively. In vivo bioluminescence imaging was used to trace tumor progression, and xenografts with SVIP expressing GBM cells displayed a significant suppression in tumor growth (Fig. 6A–C, Supplementary Fig. 4A), and SVIP overexpression resulted in higher survival rate and less weight loss of mice (Fig. 6D, E). HE staining confirmed that tumor was successfully implanted (Fig. 6F). The results of immunohistochemistry (IHC) showed that as the SVIP expression increased, the intensity of IGFBP-2 staining was decreased (Fig. 6G, Supplementary Fig. 5A, B). As demonstrated by ELISA, human IGFBP-2 (expressed solely by LN229 tumors, not by the mice) was notably reduced in mice bearing SVIP-overexpressing tumors compared to the control group (Fig. 6H). These findings collectively indicate that SVIP overexpression inhibits GBM progression and reduces IGFBP-2 production in vivo.

## Discussion

PTEN is a very important tumor suppressor. A partial loss of PTEN function has a dramatic effect on tumors [6]. According to current reports, PTEN mainly plays a role as negative regulator of PI3K/AKT signaling pathway [33]. The reduction, deletion and mutation of PTEN expression ultimately led to the activation of PI3K/AKT oncogenic pathway, which is a very common event in GBM [34]. In our study, PTEN mutations in glioma were analyzed in TCGA and other databases (Supplementary Fig. 6). R130Q mutation located in its the phosphatase functional domain with the highest mutation frequency (Supplementary Fig. 6C). Moreover, in low-grade gliomas, the mutation or deletion rate of PTEN was only 4%, whereas in high-grade gliomas (WHO III & IV), the mutation or deletion frequency of PTEN increased up to 25%. In GBM (WHO IV), the PTEN mutation frequency was 30%, and about 55% was single-allelic deletion of its gene, so only 15% patients were wild-type without mutation or deletion [6]. It is important to note that in the vast majority of cases, PTEN shows a wild-type state without mutation in gliomas of all grades (Supplementary Fig. 6A, B). Thus, a rescue of PTEN from proteosome degradation may be beneficial to prognosis of glioma patients.

**Fig. 7:**
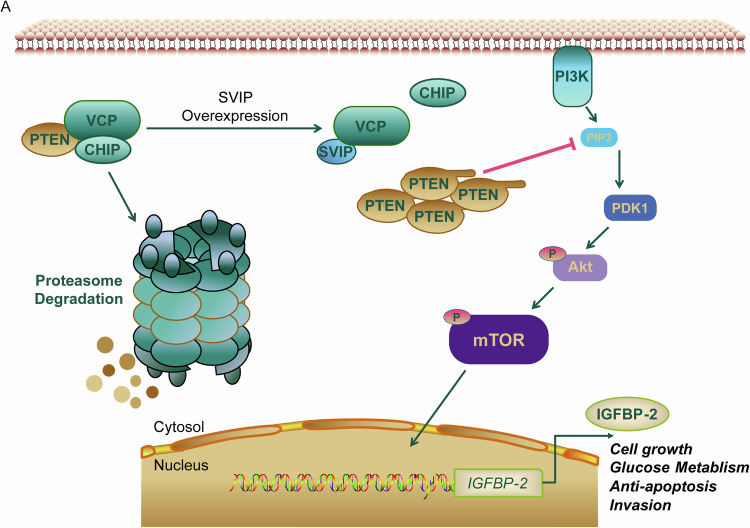
Graphical Abstract. **A** The schematic diagram illustrates the mechanism by which SVIP regulates IGFBP-2 through the PTEN/AKT/mTOR axis, thereby influencing glioblastoma progression.

According to existing studies, SVIP is theoretically of higher affinity to VCP/p97 and can play a competitive role against STUB1/CHIP [16–18]. We have demonstrated that this competitive relationship determines the expression of PTEN in GBM cells (Figs. 4 and 5), and proposed a reasonable signal axis, SVIP/PTEN/PI3K/AKT/mTOR/IGFBP-2. In the present study, decreased SVIP expression and increased STUB1/CHIP expression in GBM lead to imbalanced PTEN expression. Extensive ubiquitination and degradation of the PTEN^wt^ leads to activation of PI3K/AKT/mTOR pathway. Eventually, the elevated secretion of IGFBP-2 leads to malignant progression of GBM (Fig. 7A).

IGFBP-2 is highly expressed in many cancers, including glioma [35–37]. Previous studies have shown that overexpression of IGFBP-2 promotes glioma cell migration and invasion through activation of matrix metalloproteinase 2 protein and integrin pathways [26, 38–40]. Its higher expression is associated with malignant progression of glioma and poor survival in glioma patients. Pre-operative plasma IGFBP-2 levels were quite different between patients with high-grade glioma, low-grade glioma, and healthy individuals. Most important, high IGFBP-2 expression was statistically correlated with GBM recurrence and shorter DFS (Disease-Free Survival). In addition, plasma IGFBP-2 level increased significantly after GBM recurrence compared with pre-surgery or after two cycles of adjuvant chemotherapy. Beyond, there is a correlation between recurrence and the change in patients’ plasma levels of IGFBP-2 before and after recurrence [41]. In addition to our knowledge, we found one of the molecular mechanisms of SVIP-controlled PTEN^wt^-dependent IGFBP-2 regulation in glioma, which provides the basis for IGFBP-2 as a marker indicating PTEN status, tumor progression, recurrence and survival of high-grade glioma.

In previous studies, the function of SVIP was mainly manifested in regulating protein degradation during autophagy, however, we demonstrated that SVIP plays a key role in influencing the degradation of PTEN. SVIP acts as an autophagy regulator, which enables the colocalization of VCP/p97 with lysosomes [42] and up-regulates lipidation of autophagy marker LC3II and autophagy receptor protein p62 [31]. It also regulates the transcription factor EB (TFEB) by competing with STUB1/CHIP for VCP/p97 binding [30]. To our knowledge, mTOR (mammalian target of rapamycin) function is closely related to autophagy. mTOR is included in two distinct signal complexes, mTORC1 and mTORC2. Autophagy is inhibited by mTORC1 via phosphorylating TFEB at Ser142 and Ser211 [43]. In our study, surprisingly, overexpression of SVIP^wt^ significantly inhibited the activation of AKT/mTOR pathway and increased the expression of autophagy related genes, but SVIP^VIM^ had no effect as it lost its ability to bind to VCP/p97 (Fig. 4F, G). Therefore, the former-reported autophagy activating effect of SVIP may be partly due to stabilization of PTEN by forming of SVIP-VCP complex.

In vivo experiments showed that plasma IGFBP-2 supposed to be an unfavorable prognostic factor. High IGFBP-2 level was related to rapid tumor growth and shorter survival time (Fig. 6) indicating low expression of SVIP and high expression of STUB1 in GBM cells.

To sum up, our study indicates that the SVIP/PTEN/IGFBP-2 axis (Fig. 7) plays a crucial role in GBM progress. As a new diagnosis and treatment target for glioma, this axis is worthy of further development and research.

## Materials and methods

### Clinical sample

All tissue samples for immunohistochemical staining and plasma samples for ELISA detection were from Anhui Provincial Hospital (South District), Hefei City, Anhui Province, China. The research protocol and the collection of tissue specimens were approved by the Biomedical Research Ethics Professional Committee of Anhui Provincial Hospital (South District). Tumor tissue and non-tumor brain tissue samples were collected from pathologically confirmed glioma patients and brain trauma patients receiving surgical treatment in Anhui Provincial Hospital (South District), and blood samples were collected from pathologically confirmed GBM patients and physical examination population from March 2021 to June 2022.

### Western Blot (WB)

RIPA cell lysis buffer with phosphatase inhibitors (Selleck, USA) is used to extract total protein. The samples were separated on SDS-PAGE gel, and then transferred to methanol activated PVDF membrane (Millipore, USA) for overnight incubation of the first antibody. Anti-SVIP (1:1000) was purchased from Abcam (USA). Anti-STUB1 (1:1000) and anti-IGFBP-2 (1:500) antibodies were purchased from Proteintech (China). Anti-PTEN (1:1000), anti-AKT (1:1000), anti-Phospho-Akt (Ser473) (1:1000), anti-mTOR (1:500), anti-Phospho-mTOR (Ser2448) (1:500), anti-4EBP-1(1:1000), anti-Phospho-4EBP-1 (Thr37/46) (1:1000) were purchased from Cell Signaling Technology (USA). Anti-VCP/p97 (1:1000) was purchased from Santa Cruz Biotechnology (USA). Anti-ACTB (1:1000) was purchased from Sangon Biotech (China). HRP-labeled secondary antibody (Sangon Biotech, China) incubate at room temperature for 1 h. The protein bands were visualized using a chemiluminescence reagent (ECL) kit (Biosharp, China). The original uncropped images of western blot membranes are provided in Supplementary Data.

### Immunoprecipitation (IP)

IP buffer (1 M Tris-HCl, 150 mM NaCl, 0.5 mM EDTA, 2 mM MgCl_2_ in ddH_2_O) with protease inhibiter cocktail (Roche, Switzerland) was used to lyse cells. According to the manufacturer’s instructions, BCA protein quantitative detection reagent is used for protein quantification (Sangon Biotech). Invert anti-PTEN (1:1000) (Cell Signaling Technology), anti-VCP/p97 (1:1000) (Santa Cruz Biotechnology) antibody and protein at 4 °C for 4–6 h. Seal Protein G Dynabeads (Thermo, USA) with 5% BSA for 1 h. Add antibody protein mixture, turn over and shake for 8 h. After the IP buffer and cocktail wash the magnetic beads for three times, centrifugate and collect the magnetic beads. 1× The loading buffer was added and detected protein expression by SDS-PAGE and immunoblotting.

### Immunofluorescence (IF)

The immunofluorescence staining kit was purchased from ImmunoWay Biotechnology Company. LN229 cells were seeded into a confocal dish (Biosharp, China). Stain and antibody stripping were performed according to the instructions provided by the fluorescent detection kit manufacturer. Dilute the antibody with 5% BSA in a ratio of 1:250, anti-VCP/p97 (Santa Cruz Biotechnology), anti-STUB1 (Proteintech), anti-SVIP (Abcam), anti-PTEN (CST). After incubation at 4 °C overnight, wash with PBS for 3 times. The immunofluorescence staining was performed using 488-labeled Tyramide, 594-labeled Tyramide, and 647-labeled Tyramide to label PTEN, VCP/p97, and STUB1 (SVIP), respectively. Immunofluorescently labeled cells were observed and photographed using a laser scanning confocal microscope (LSCM) (ZEISS LSM800, Germany).

### Enzyme linked immunosorbent assay (ELISA)

IGFBP-2 ELISA kit (Elabscience, China) was used to measure the IGFBP-2 level in clinical plasma samples according to the manufacturer’s instructions.

### Tumor xenograft model

Female BALB/c nude mice (4–5 weeks old) were purchased from GemPharmatech Jiang Su and raised in a laminar flow box under specific pathogen free conditions. Stereotactic injection of LN229 cells stably expressing SVIP-Luc and control-Luc into mouse brain, 5–6 × 10^5^ cells/mouse. Bioluminescence imaging was used to monitor the growth of intracranial tumors. When the body weight of a mouse decreased by more than 30%, the mouse was considered to death. After the mice were sacrificed, brain tissues and plasma were taken for subsequent experimental studies. All animal studies were approved by the Institutional Animal Care and Use Committee of the First Affiliated Hospital of USTC.

### Supplementary information

SUPPLEMENTAL MATERIAL

original data files

## Data Availability

The data that support the findings of this study are available from the corresponding author upon reasonable request.
