# Supplementary material for: SVIP reduces IGFBP-2 expression and inhibits glioblastoma progression via stabilizing PTEN
Source: Cell Death Discov. 2024 Aug 13;10:362. doi: 10.1038/s41420-024-02130-z (PMC11322382; doi:10.1038/s41420-024-02130-z)
Supplement: Supplementary file 1 — SUPPLEMENTAL MATERIAL [file 41420_2024_2130_MOESM1_ESM.docx]

**Inventory of supplementary information**

**Supplementary Materials and Methods**

**Cell culture**

Human GBM cells (U87MG, LN229) were purchased from American Type Culture Collection (ATCC). These cells were authenticated using an STR assay (Beijing Genomics institution). Cells were cultured in DMEM containing 10% FBS (Clark Bioscience, Australia) and maintained in a humidified incubator at 37 °C in an atmosphere of 5% CO_2_. (All experiments were performed with mycoplasma-free cells)

**Cell transfection**

SVIP-pcDNA3.1, STUB1/CHIP-pcDNA3.1, IGFBP2-pcDNA3.1, PTEN-WT-GFP-pcDNA3.1, PTEN-R130Q-GFP- pcDNA3.1 and empty vector were provided by FENGHUIBIO (Wuhan, China). sh-IGFBP2 was obtained from Ribobio (China). Lipofectamine2000 (Invitrogen, USA) were used to transfect cells.

**RNA extraction and quantitative real-time PCR assays**

Total RNA was extracted from GBM cell lines (U87MG, LN229) using TRIzol reagent (Invitrogen, USA) according to the manufacturer’s protocol. RNA (0.5μg) was reverse transcribed using a GoScript reverse transcription system (Promega, Beijing) and the corresponding primers. Real-time PCR analyses were performed using Trans Start Top Green qPCR SuperMix (+Dye II) (TransGen) on an ABI Q5 sequence detection system (Applied Biosystems); Normalize the threshold cycle of each gene with the threshold cycle (Ct value) of GAPDH, and use 2 − ΔΔ Ct for quantitative analysis. All primers were synthesized by Sangon Biotech. The primer sequences used include IGFBP-2: F, 5’-GACAATGGCGATGACCACTCA-3’ and R, 5’-GCTCCTTCATACCCGACTTGA-3’; GAPDH: F, 5’-CTTCATTGACCTCAACTACATGG-3’ and R, 5’-CTCGCTCCTGGAAGATGGTGAT-3’; ATG5: F, 5’-ATGTGCTTCGAGATGTGTG-3’ and R, 5’- GTGTGCCTTCATATTCAAACC-3’; ATG7: F, 5’-ACCCAGAAGAAGCTGAACGA-3’ and R, 5’-CTCATTTGCTGCTTGTTCCA-3’; LC3B: F, 5’-ATGCCGTCGGAGAAGACC-3’ and R, 5’-CACTGACAATTTCATCCCG-3’; PTEN: F, 5’-TCCCAGACATGACAGCCATC-3’ and R, 5’- TGCTTTGAATCCAAAAACCTTACT-3’.

**CCK-8 cell viability assay**

After transfection U87MG and LN229 cells, 4×10^3 cells / well were seeded into 96-well plate. According to manufacturer’s protocol, the cell proliferation was detected with the CCK-8 reagent (TransGen).

**Wound Healing assay**

U87MG or LN229 cells, 1.0 × 10^6 cells / well, were seeded into 6-well plate. After cell fusion, scratch the single-layer cells with the tip of the pipette gun, then rinse the cells with PBS for three times, and culture the cells with the medium containing 2% fetal bovine serum. After 0, 24 and 48 h incubation, the cells were photographed with an inverted microscope (Olympus, Japan) to detect the cell migration.

**Transwell cell migration assay**

The migration abilities of U87MG and LN229 cells were evaluated using Transwell chambers (8μm pore size, 24-well insert; Costar). Following transfection, cells were cultured for 48 hours (0.8 × 10^4) and seeded into the upper chamber using serum-free medium, while medium containing 10% fetal bovine serum (FBS) was added to the lower chamber. The number of cells that migrated into the lower chambers was enumerated using an inverted microscope (Olympus).

**EdU cell proliferation assay**

The EdU Cell Proliferation Assay Kit was purchased from Beyotime (China). Cultivate an appropriate number of cells in a 24-well plate. After overnight incubation for cells to recover to a normal state, proceed with cell transfection and continue incubation for 48 hours. Prepare the EdU cell labeling solution following the manufacturer's instructions and continue incubation for 2 hours. After labeling the cells with EdU, fix them with 4% paraformaldehyde and permeabilize the cell membranes using PBS containing 0.3% Triton X-100. Prepare the Click reaction solution according to the manufacturer's instructions and incubate at room temperature in the dark for 30 minutes. Finally, conduct nuclear staining and detect cell proliferation using a fluorescence microscope at 647nm.

**Lentivirus infection**

The SVIP CDS region was synthesized by Crius Biotechnology (China) and inserted into the Plv4ltr Puro CMV retroviral vector. In 293T cells, the SVIP stable plasmid and three auxiliary plasmids were transfected according to the instructions of lipo2000. The lentivirus produced infected LN229 cells for 48 hours. Cells were screened with puromycin (2μg/mL) to obtain stable overexpression of SVIP cell lines.

**Statistical analysis**

All statistical analyses were performed using GraphPad Prism 8.0 software. T-test and analysis of variance were used to evaluate the significance of the differences between groups. Unless otherwise stated, the data are expressed in SD ± mean. P<0.05 was considered statistically significant. (ns, non-significant difference; *p < 0.05; **p < 0.01; ***p < 0.001)

**Immunohistochemistry (IHC)**

Formalin-fixed-paraffin-embedded human glioma tissues of different grades (Ⅰ and Ⅱ are low grade, Ⅲ and Ⅳ are high grade) and non-tumor brain tissues were dewaxed. The endogenous peroxidase activity was blocked with 3% H_2_O_2_ and repaired in sodium citrate buffer (pH 6.0). After natural cooling, block with 10% goat serum and 1% Triton X-100. Incubate with anti-SVIP (abcam), anti-IGFBP2 and anti-STUB1 (Proteintech), antibodies diluted in 1:200-500, and incubate overnight at 4 ° C. The antigen antibody complex was detected with the substrate of diaminobenzidine (DAB) (ZSGB-BIO, China), and then the nucleus was counterstained with hematoxylin (China). Representative images were obtained with an inverted microscope (Olympus, Japan). Five samples were taken from each sample for positive rate analysis, and the sample size of different grade tissue sections was more than 3. The evaluation of SVIP, IGFBP2 and STUB1 expression was based on the staining intensity and the percentage of positive cells in the total number of tumor cells.

**Supplementary Figures**

**
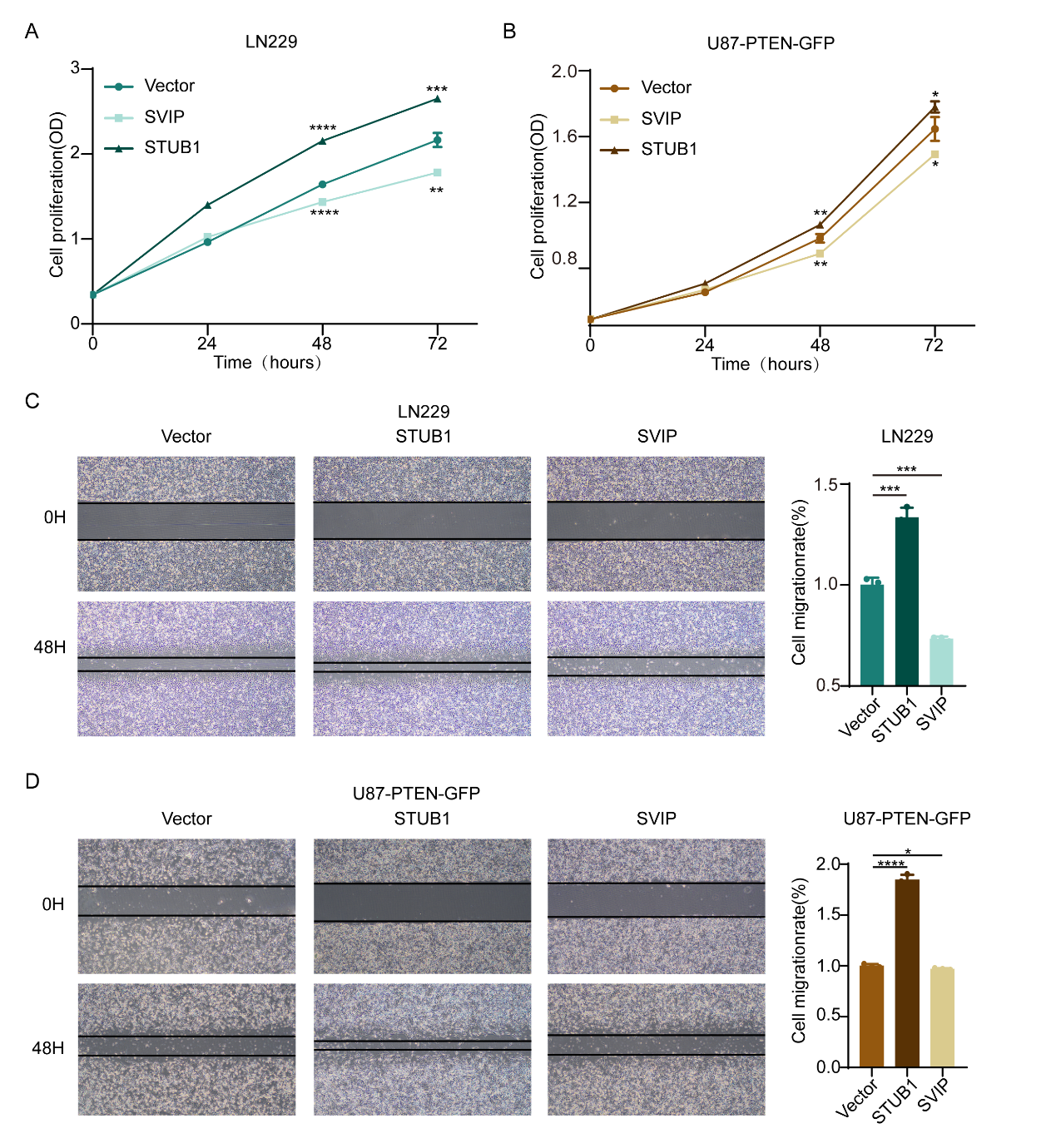
**

**Supplementary Fig.1**

**A**. LN229 cells were transfected with STUB1 or SVIP overexpression plasmids, respectively, and cell viability was assessed using the CCK8 assay. **B**. U87-MG cells were transfected with the PTEN-WT-GFP plasmid followed by either the STUB1/CHIP or SVIP overexpression plasmid, respectively. Cell viability was assessed using the CCK8 assay. **C**. LN229 cells were transfected with STUB1 or SVIP overexpression plasmids, respectively, and cell migration was assessed by wound healing experiments. **D**. U87-MG cells were transfected with the PTEN-WT-GFP plasmid followed by either the STUB1 or SVIP overexpression plasmid, respectively. Cell migration ability was assessed using wound healing experiments. Statistical analysis: data were quantified as mean ± SD, n ≥ 3, two tailed student's t test, P < 0.05, *; P＜0.01，**; P＜0.001, ***; P＜0.0001, ****.

**
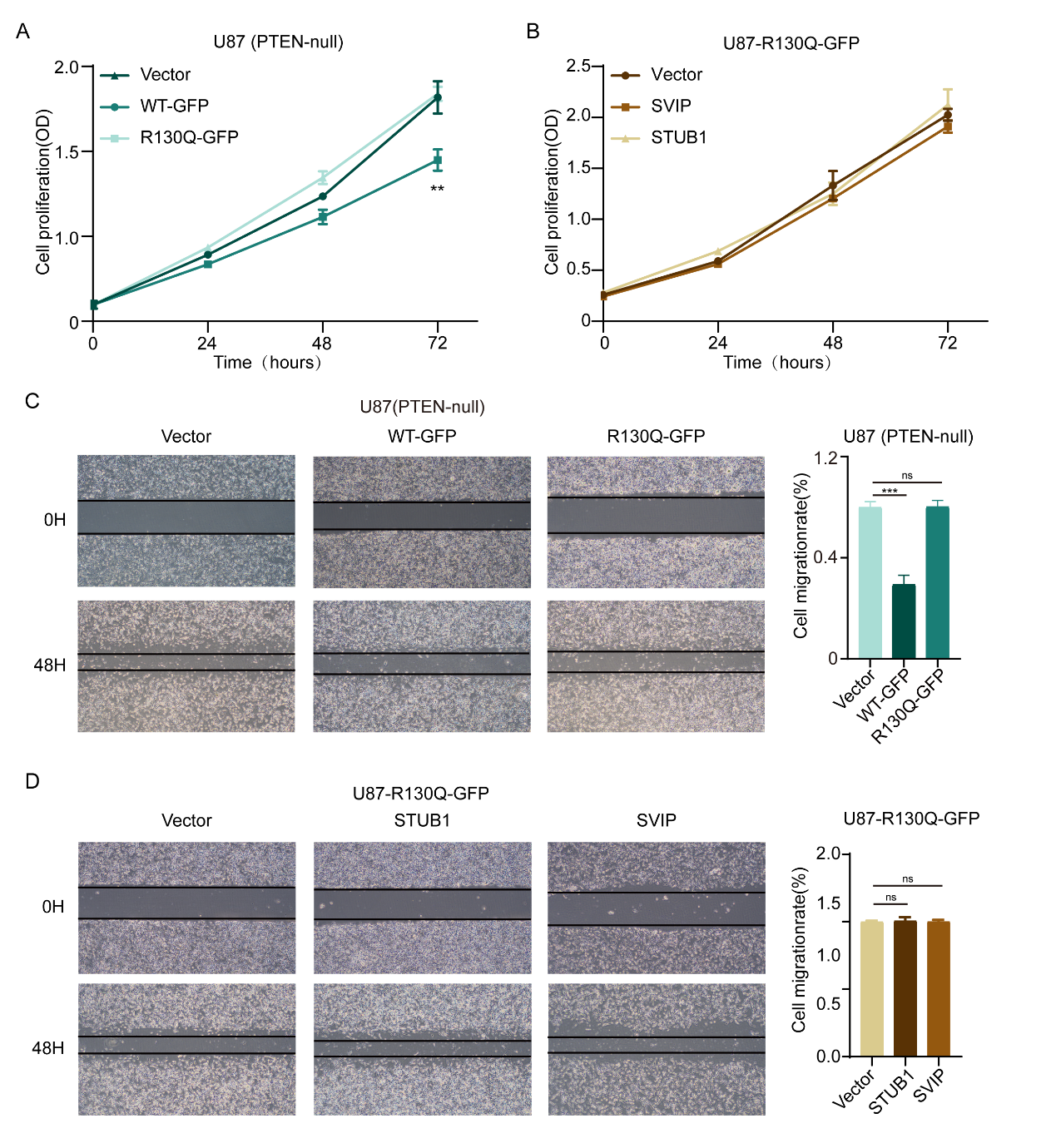
**

**Supplementary Fig.2**

**A**. U87-MG cells were separately transfected with PTEN-WT-GFP and PTEN-R130Q-GFP overexpression plasmids. Cell viability was assessed using the CCK8 assay. **B**. U87-MG cells were transfected with the PTEN-R130Q-GFP plasmid followed by either the STUB1 or SVIP overexpression plasmid, respectively. Cell viability was assessed using the CCK8 assay.

**C**. PTEN-WT-GFP and PTEN-R130Q-GFP overexpression plasmids were transfected separately in U87-MG cells, and cell migration was assessed by wound healing experiments. **D**. U87-MG cells were transfected with the PTEN-R130Q-GFP plasmid followed by either the STUB1 or SVIP overexpression plasmid, respectively. Cell migration ability was assessed by wound healing experiments. Statistical analysis: data were quantified as mean ± SD, n ≥ 3, two tailed student's t test, P < 0.05, *; P＜0.01，**; P＜0.001, ***; P＜0.0001, ****. ns, no significance.

**
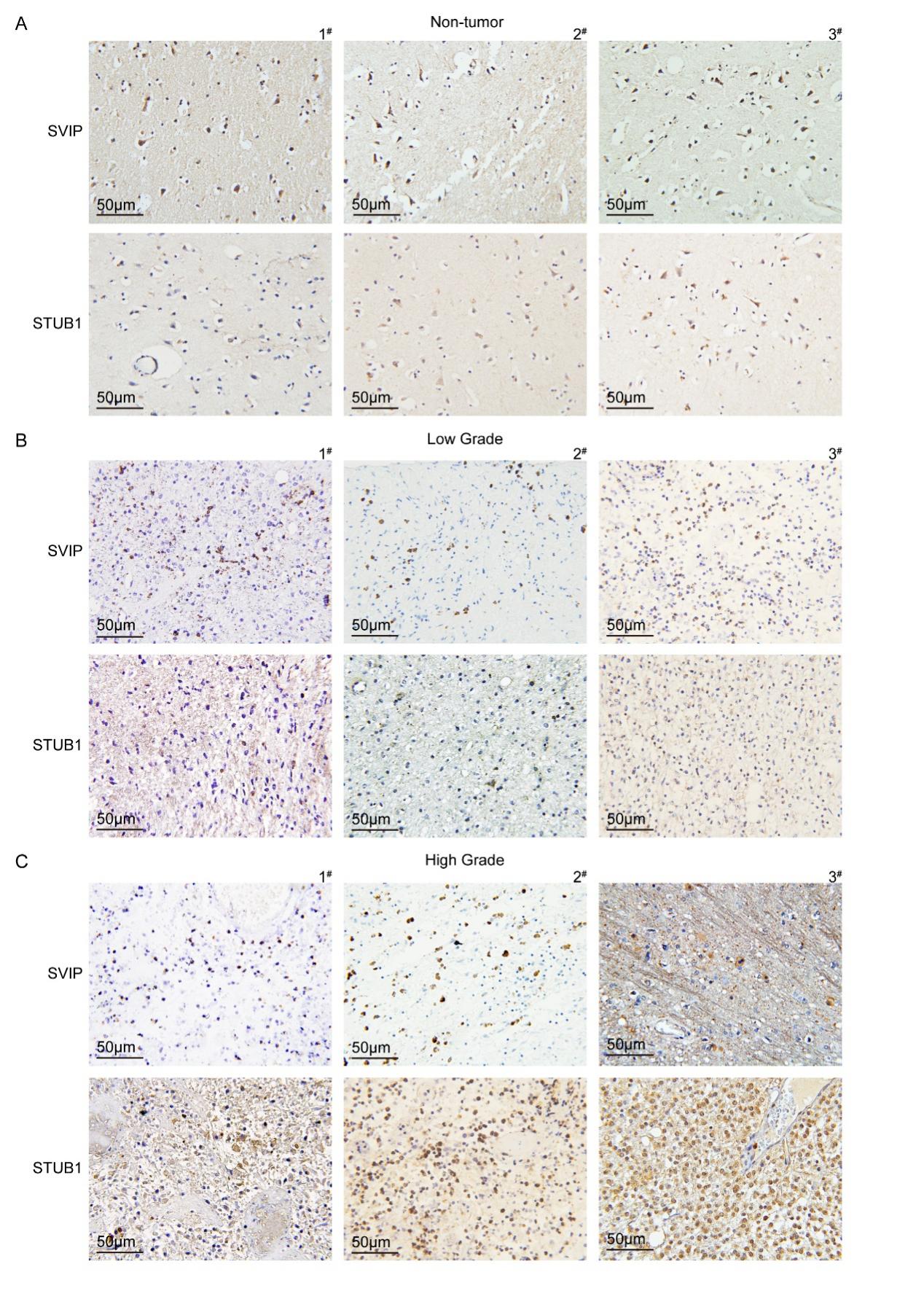
**

**Supplementary Fig.3**

**A**. The expression of SVIP and STUB1 in non-tumor brain tissue was assessed by immunohistochemistry (IHC). **B**. The expression of SVIP and STUB1 in low-grade (WHO grades I and II) glioma tissues was detected by IHC. **C**. The expressions of SVIP and STUB1 in high-grade (WHO grades III and IV) glioma tissues were detected by IHC. All images were captured at 40× magnification.

**
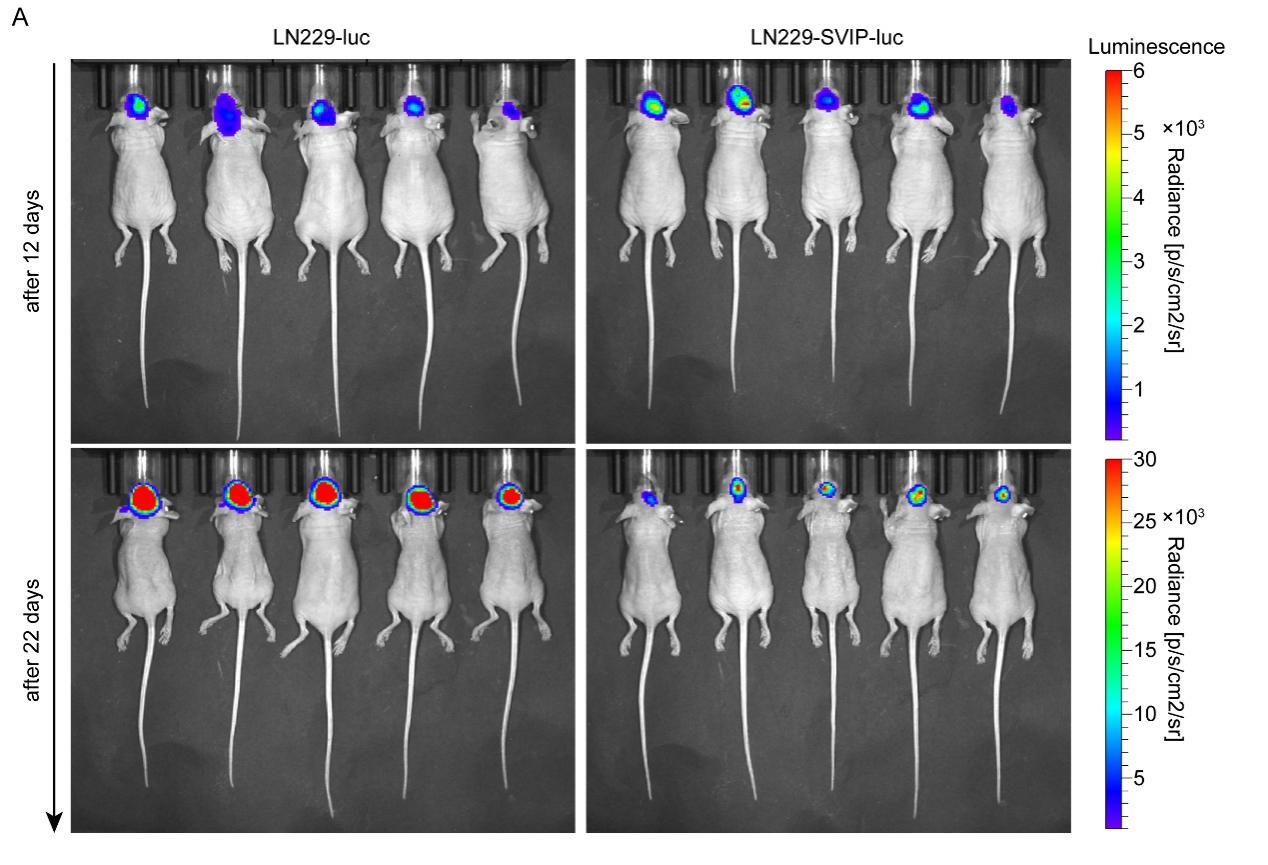
**

**Supplementary Fig.4**

**A**. Bioluminescence images of nude mice.

**
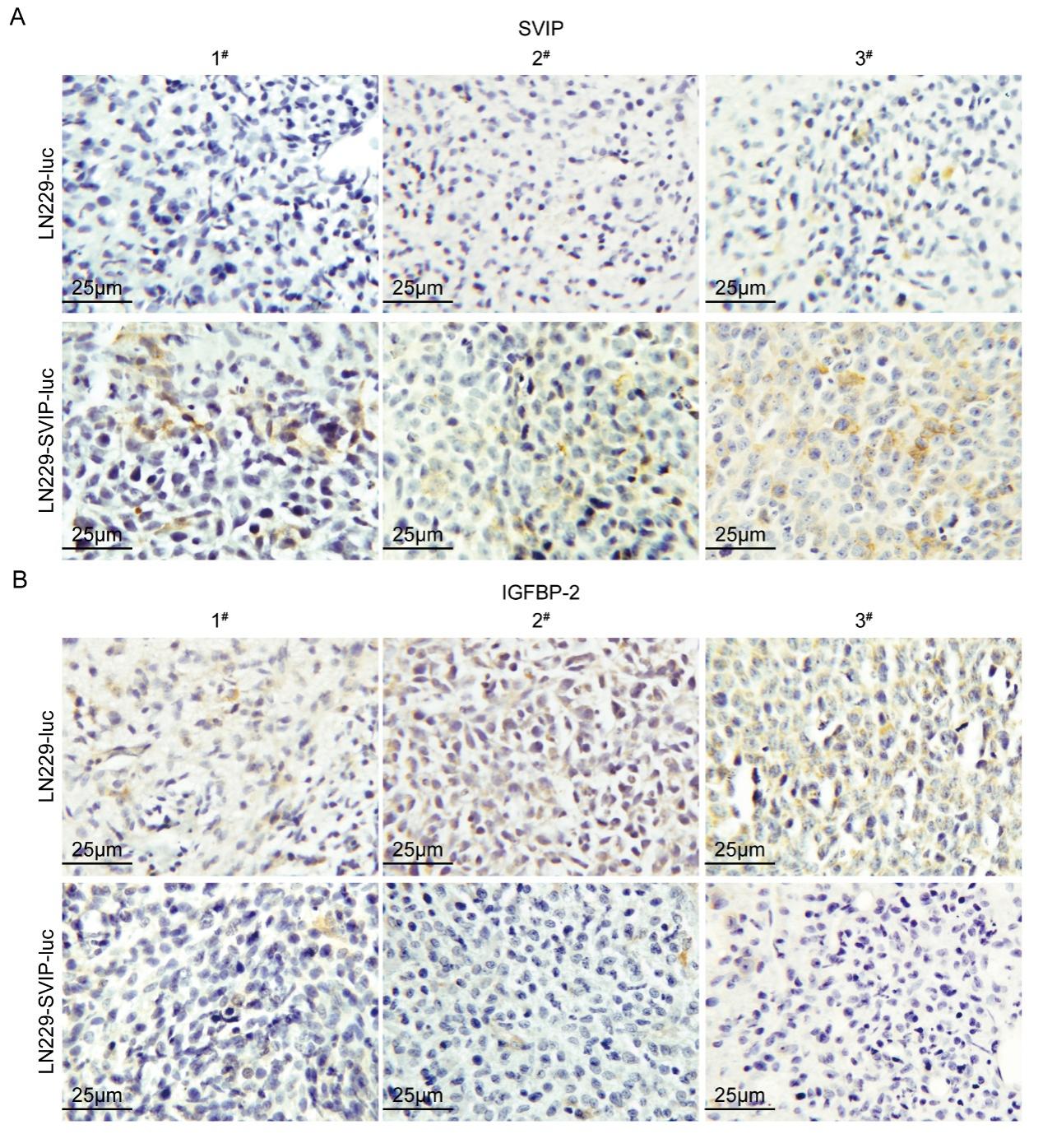
**

**Supplementary Fig.5**

**A**. Immunohistochemistry (IHC) was performed to detect the expression of SVIP in murine brain paraffin sections. **B**. Immunohistochemistry (IHC) was performed to detect the expression of IGFBP-2 in murine brain paraffin sections. All images were captured at a magnification of 40× and subsequently enlarged by one-fold.

**
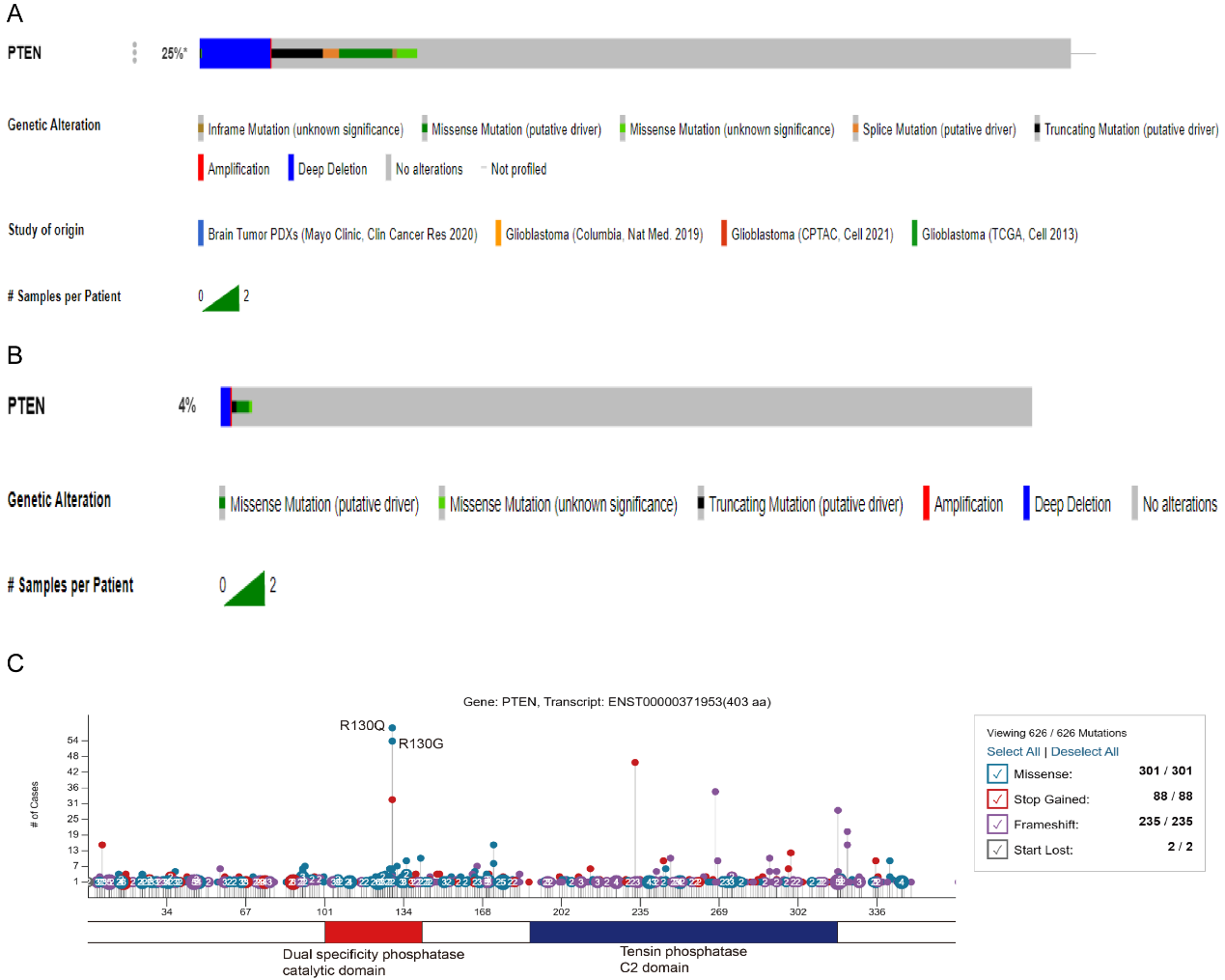
**

**Supplementary Fig.6**

**A**. Analysis of PTEN mutation rates in low-grade gliomas using TCGA database. **B**. Analysis of PTEN mutation rates in glioblastoma using TCGA database. **C**. Mutation site analysis of PTEN using TCGA database. The x-axis represents the position of specific amino acids, while the y-axis indicates the mutation rate.
