# Supplementary material for: SVIP reduces IGFBP-2 expression and inhibits glioblastoma progression via stabilizing PTEN
Source: Cell Death Discov. 2024 Aug 13;10:362. doi: 10.1038/s41420-024-02130-z (PMC11322382; doi:10.1038/s41420-024-02130-z)
Supplement: Supplementary file 2 — original data files [file 41420_2024_2130_MOESM2_ESM.pdf]

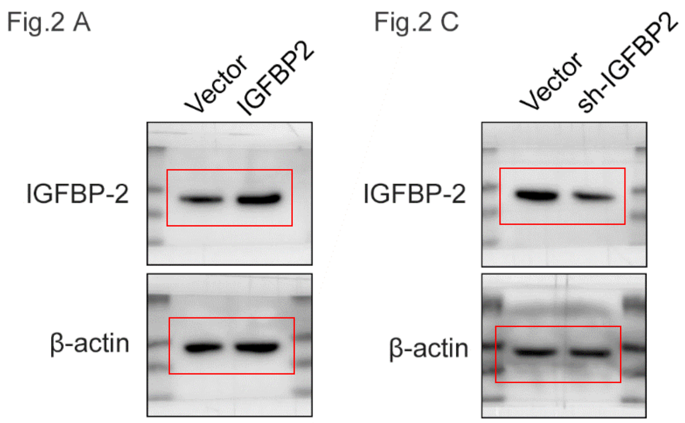

Fig.2 A&C In U87-MG cells, the efficiency of IGFBP-2 overexpression and knockdown was evaluated using Western blot.

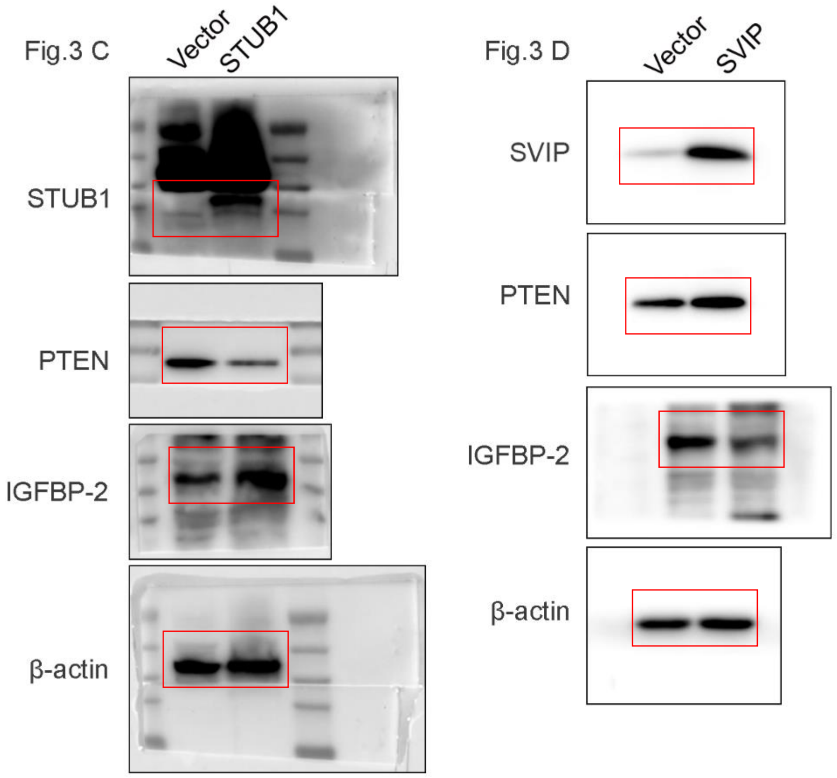

Fig.3 C-D In LN229 cells, transfection was performed with STUB1 or SVIP overexpression plasmids separately. Subsequently, Western blot analysis was conducted to assess the expression levels of STUB1, SVIP, PTEN, and IGFBP-2.

Fig.3 E

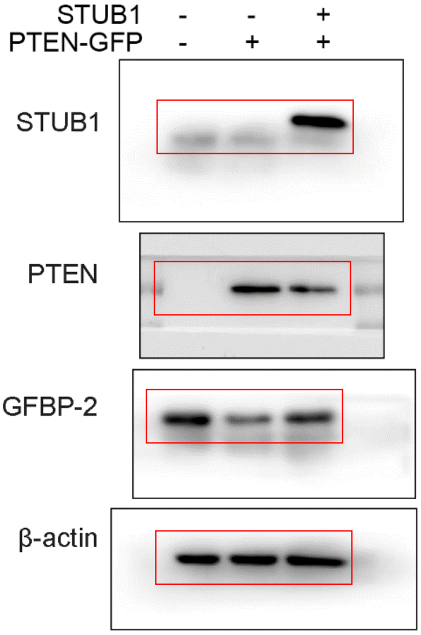

Fig.3 F

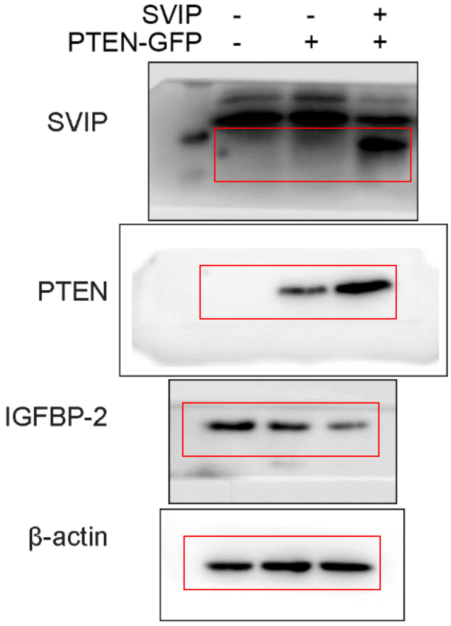

Fig.3 E U87-MG cells were co-transfected with PTEN-GFP overexpression plasmid and empty vector, PTEN-GFP and STUB1 overexpression plasmids, respectively. Western blot analysis was conducted to evaluate the protein expression levels of STUB1, PTEN, and IGFBP-2 in the U87 cells. **Fig.3 F** U87-MG cells were co-transfected with PTEN-GFP overexpression plasmid and empty vector, PTEN-GFP and SVIP overexpression plasmids, respectively. Western blot analysis was conducted to evaluate the protein expression levels of SVIP, PTEN, and IGFBP-2 in the U87 cells.

Fig.4 C

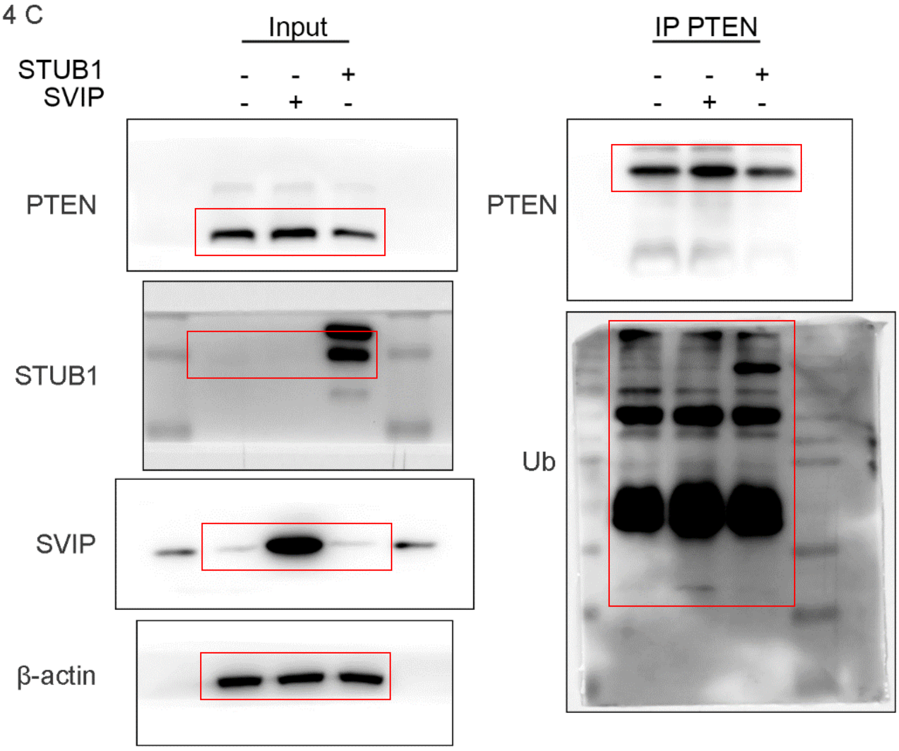

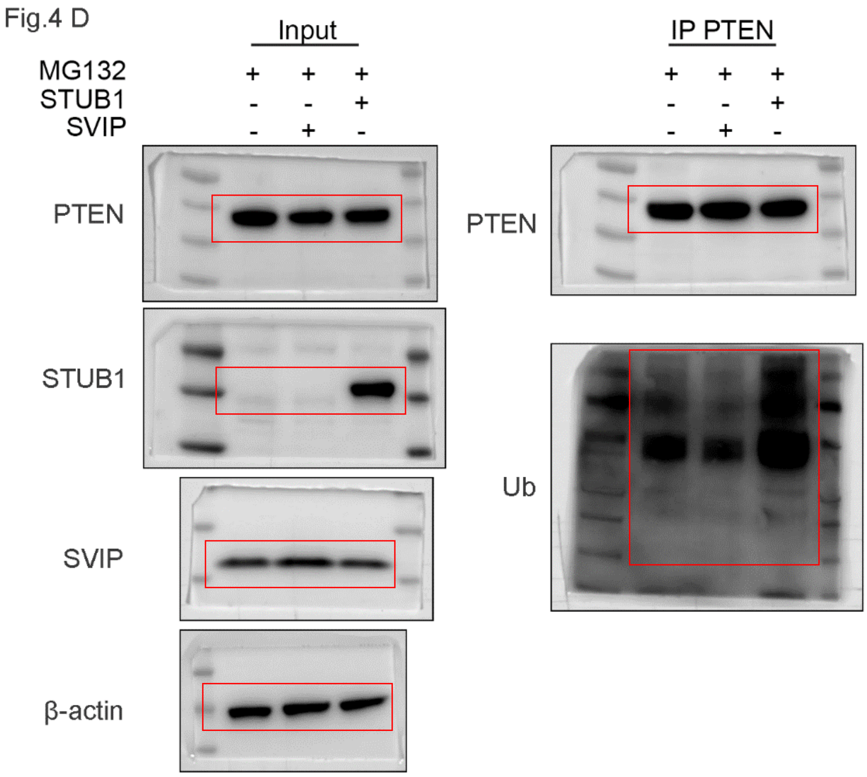

Fig.4 C-D In LN229 cells, plasmids overexpressing STUB1/CHIP and SVIP were separately transfected. After 48 hours of transfection, cells were treated with or without MG132 (50uM, TOPSCIENCE, China) for 2 hours. Immunoprecipitation (IP) was performed using an anti-PTEN antibody, followed by immunoblotting (IB) with an anti-Ub antibody to assess the binding between PTEN and Ub.

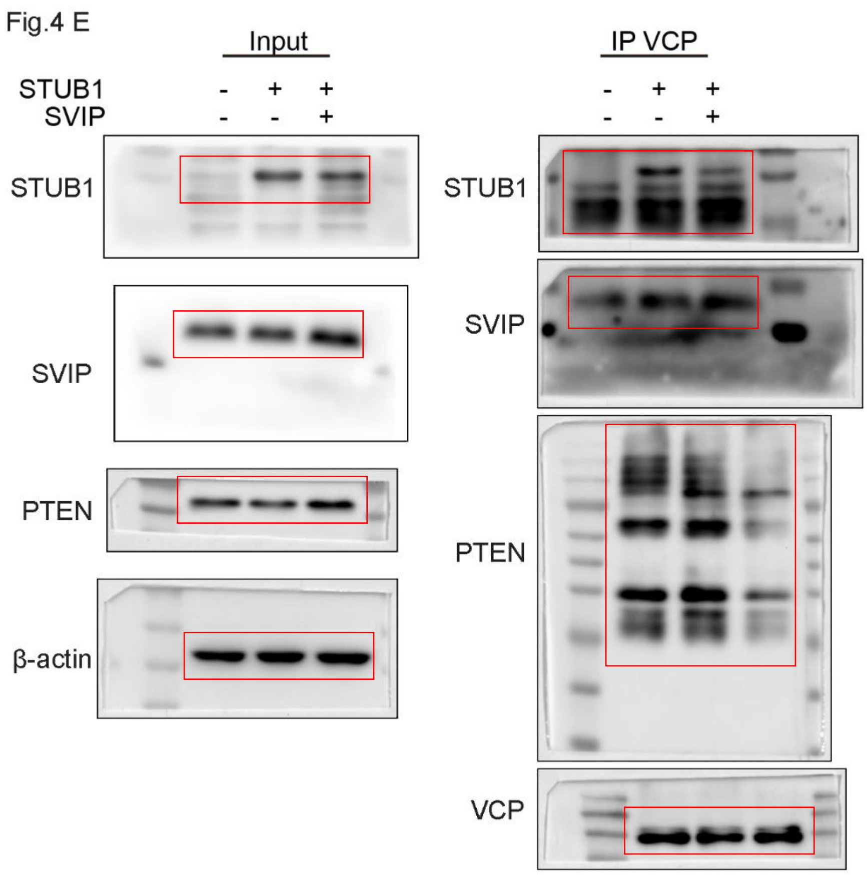

Fig.4 E In LN229 cells, co-transfection was conducted using either the STUB1/CHIP overexpression plasmid and empty vector, or the STUB1 and SVIP overexpression plasmid. Immunoprecipitation (IP) was carried out using an anti-VCP antibody, followed by immunoblotting (IB) with anti-STUB1, anti-SVIP, and anti-PTEN antibodies, respectively, to detect the interaction between VCP and PTEN.

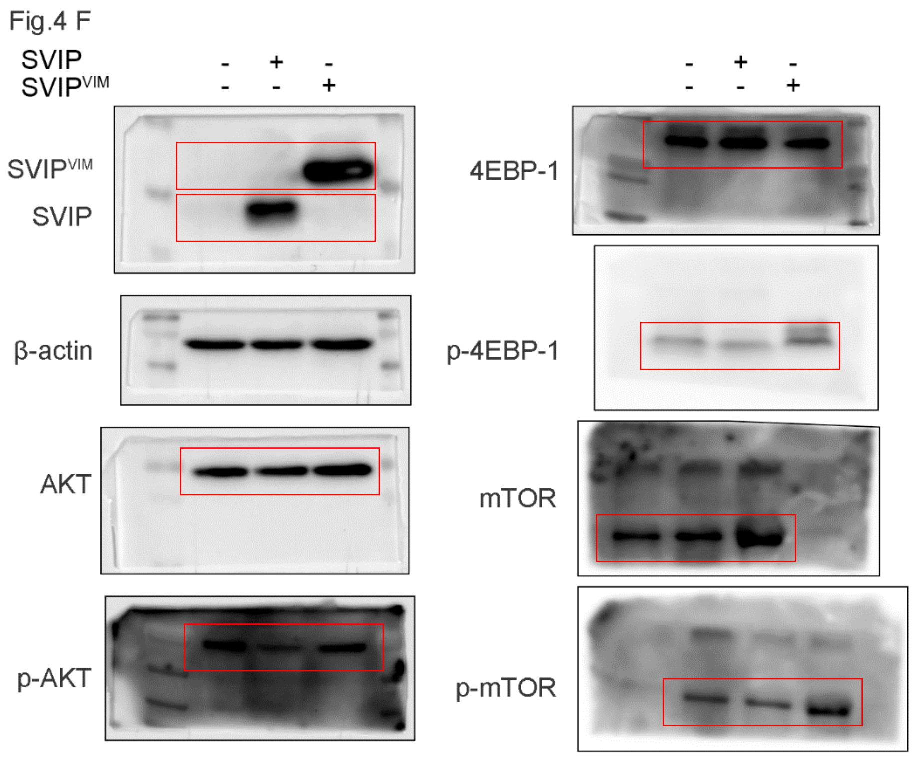

Fig.4 F Transfect LN229 cells with SVIP and SVIP^{VIM} overexpression plasmids, then assess Akt, mTOR, and 4EBP-1 expression and phosphorylation levels via Western blot.

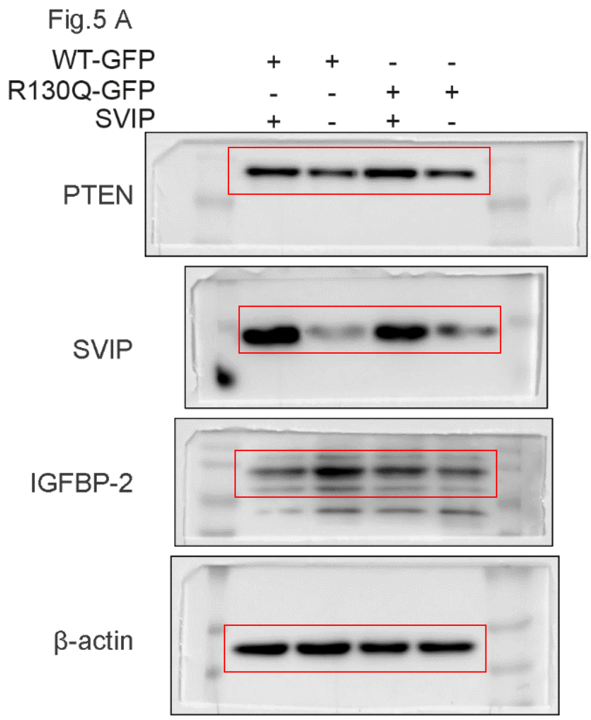

Fig.5 A Co-transfection experiments were conducted in U87-MG cells with PTEN-WT-GFP or PTEN-R130Q-GFP overexpression plasmids, along with empty vectors or SVIP overexpression plasmids. Subsequently, Western blot analysis was employed to assess the protein levels of SVIP, PTEN, and IGFBP-2.

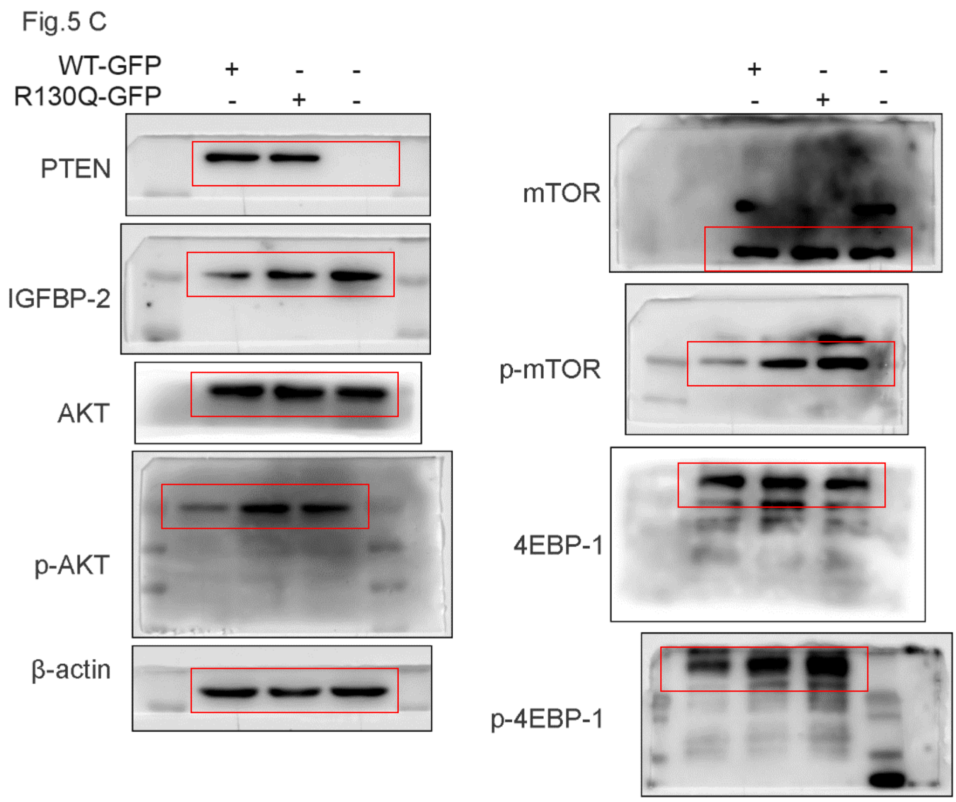

Fig.5 C PTEN-WT-GFP and PTEN-R130Q-GFP overexpression plasmids were transfected into U87-MG cells, and the protein expression levels and phosphorylation levels of Akt, mTOR, and 4E-BP1 were assessed by Western blot.

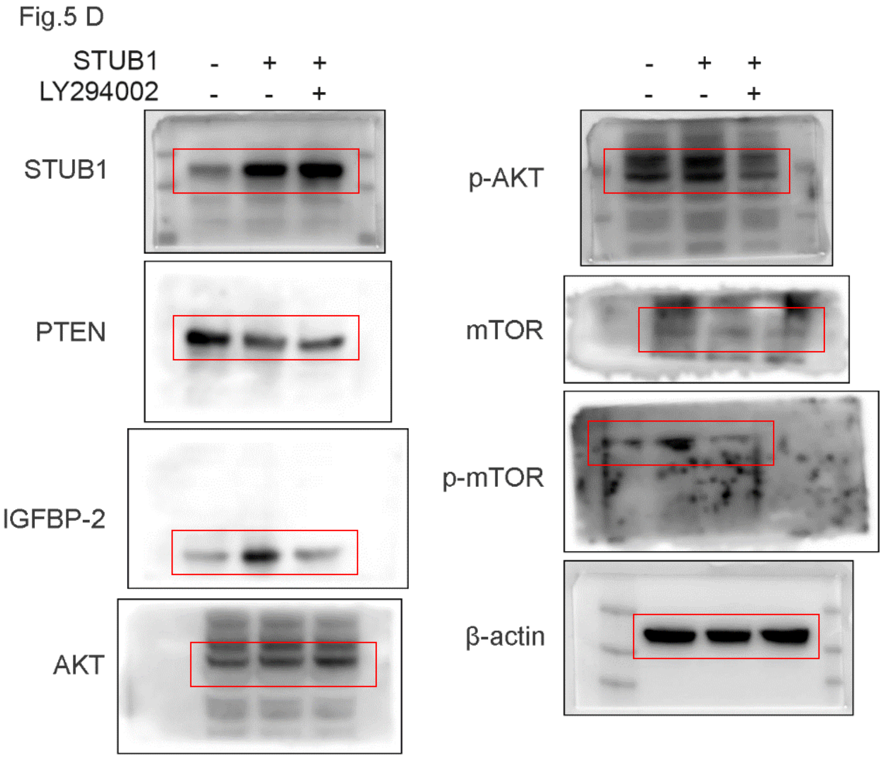

Fig.5 D The STUB1 overexpression plasmid was transfected into LN229 cells, followed by treatment with or without the LY294002 inhibitor (20μM, TOPSCIENCE, China). Subsequently, the expression levels of STUB1 and PTEN, as well as the phosphorylation levels of AKT and mTOR, were assessed by Western blot.
